# Age Related Differences in Responsiveness to Sildenafil and Tamsulosin are due to Myogenic Smooth Muscle Tone in the Human Prostate

**DOI:** 10.1038/s41598-017-07861-x

**Published:** 2017-08-31

**Authors:** Sophie N. Lee, Basu Chakrabarty, Brad Wittmer, Melissa Papargiris, Andrew Ryan, Mark Frydenberg, Nathan Lawrentschuk, Ralf Middendorff, Gail P. Risbridger, Stuart J. Ellem, Betty Exintaris

**Affiliations:** 1 Drug Discovery Biology, Monash Institute of Pharmaceutical Sciences, Melbourne, Victoria Australia; 2 Department of Anatomy and Developmental Biology, Biomedicine Discovery Institute, Monash University, Clayton, Victoria Australia; 3 TissuePath, Melbourne, Victoria Australia; 4 Department of Surgery, Monash University, Melbourne, Victoria Australia; 5 Australian Urology Associates, Melbourne, Victoria Australia; 6 Department of Surgery, Austin Health, University of Melbourne, Melbourne, Victoria Australia; 7 Justus-Liebig-University Giessen, Institute of Anatomy and Cell Biology, Giessen, Germany

## Abstract

Lower urinary tract symptoms (LUTS) due to Benign Prostatic Hyperplasia (BPH) are highly prevalent in older men, having a profound impact on patient quality of life. Current therapeutics for BPH/LUTS target neurogenic smooth muscle tone, but response is unpredictable and many patients fail to respond. Spontaneous myogenic tone is another component of smooth muscle contractility that is uncharacterized in human prostate. To better understand and improve the predictability of patient response, we defined myogenic contractility using human prostate specimens and examined the effect of existing therapeutics. We show that myogenic activity is present in the human prostate with the frequency of contractions in transition zone (TZ) specimens from BPH diagnosed patients approximately 160% greater than matched controls. α1-adrenoreceptor antagonists (Tamsulosin) and PDE5 inhibitors (Sildenafil) both significantly reduced myogenic contractile parameters, including frequency, with notable interpatient variability. Tamsulosin was more effective in older patients (R^2^ = 0.36, *p* < 0.01) and men with larger prostate volumes (R^2^ = 0.41, *p* < 0.05), while Sildenafil was more effective in younger men (R^2^ = 0.45, *p* < 0.05). As myogenic tone is significantly increased in BPH, therapeutics targeting this mechanism used with reference to patient characteristics could improve clinical outcomes and better predict patient response.

## Introduction

Benign Prostatic Hyperplasia (BPH) is a complex, non-malignant, and highly prevalent disease that affects ageing men^1^. This disease affects 50% of men over 50 and 90% of men over 80, resulting in severe lower urinary tract symptoms (LUTS) that have an enormous impact on a patients quality of life^2^. This problem is compounded by the aging population^3^, and is further exacerbated by the limited efficacy of current front-line therapeutics and the unpredictable nature of patient response to these drugs. Consequently, there is an urgent need to better understand the physiology and etiology of BPH in order to improve patient treatment and outcome^4^.

There are two distinct components that underlie the etiology of BPH, a static and a dynamic component^5^. The static component consists of stromal hyperplasia or hypertrophy, leading to an increased prostate size. The dynamic component is characterized by changes in smooth muscle contractility, notably tone. Pharmacotherapies targeting smooth muscle tone, notably α1_A_-adrenoreceptor antagonists (alpha blockers)^4^ and Phosphodiesterase-5 inhibitors (PDE5-Is), are currently used for the treatment of BPH and alleviation of LUTS. However, both pharmacotherapies are limited due to their unpredictable efficacy in a given patient, insufficient relief of symptoms, and adverse side-effects^6, 7^. A retrospective analysis into the discontinuation of alpha blockers found that of an initial study population of 13,474 men, 47.1% terminated treatment within the first year, with only 30.1% of men compliant with treatment after 12 months^8^. Phosphodiesterase-5 inhibitors (PDE5-Is) promote smooth muscle relaxation through blocking the degradation of cGMP, and were proposed as a therapy for BPH in 2007 after men with erectile dysfunction reported relief of BPH symptoms while on PDE5-Is^9, 10^. Preclinical data indicates that PDE5-Is act on the lower urinary tract at multiple sites and via multiple mechanisms^7^, although a definitive mechanism of action in the human prostate has yet to be elucidated.

Smooth muscle tone specifically refers to the sustained state of contraction within a muscle and can be further separated into neurogenic and myogenic components^11^. Neurogenic tone has been well described in the human prostate, and is mediated by release of neurotransmitters from the autonomic nervous system, notably during ejaculation^12^. Myogenic tone is the inherent and spontaneous rhythmicity of the tissue, which is mediated independently of stimulus from the autonomic nervous system. Myogenic tone is well characterized in other lower urinary tract organs, notably the bladder^13^, where overactive bladder (OAB) is now recognized to be driven by changes in myogenic contractility^14^, and several therapies have been developed to target this^15^. Despite extensive characterization of neurogenic contractility of the prostate, the myogenic tone of the human prostate has yet to be characterized.

In this study we demonstrate that myogenic tone is present in the human prostate by utilizing primary prostate specimens from a clinically diverse cohort of men. We show that the frequency of myogenic tone is significantly increased in men with clinical/symptomatic BPH. Both existing (alpha blockers; tamsulosin) and emerging (PDE5-Is; sildenafil) therapeutics attenuated myogenic tone, with interpatient variability in responsiveness significantly correlated with age and prostate volume. Importantly, these data also show that clinical parameters could be used to predict efficacy and thus improve patient response and outcome.

## Results

### Spontaneous myogenic tone exists in the human prostate and is significantly different in Transition versus Peripheral zones

**Figure 1:**
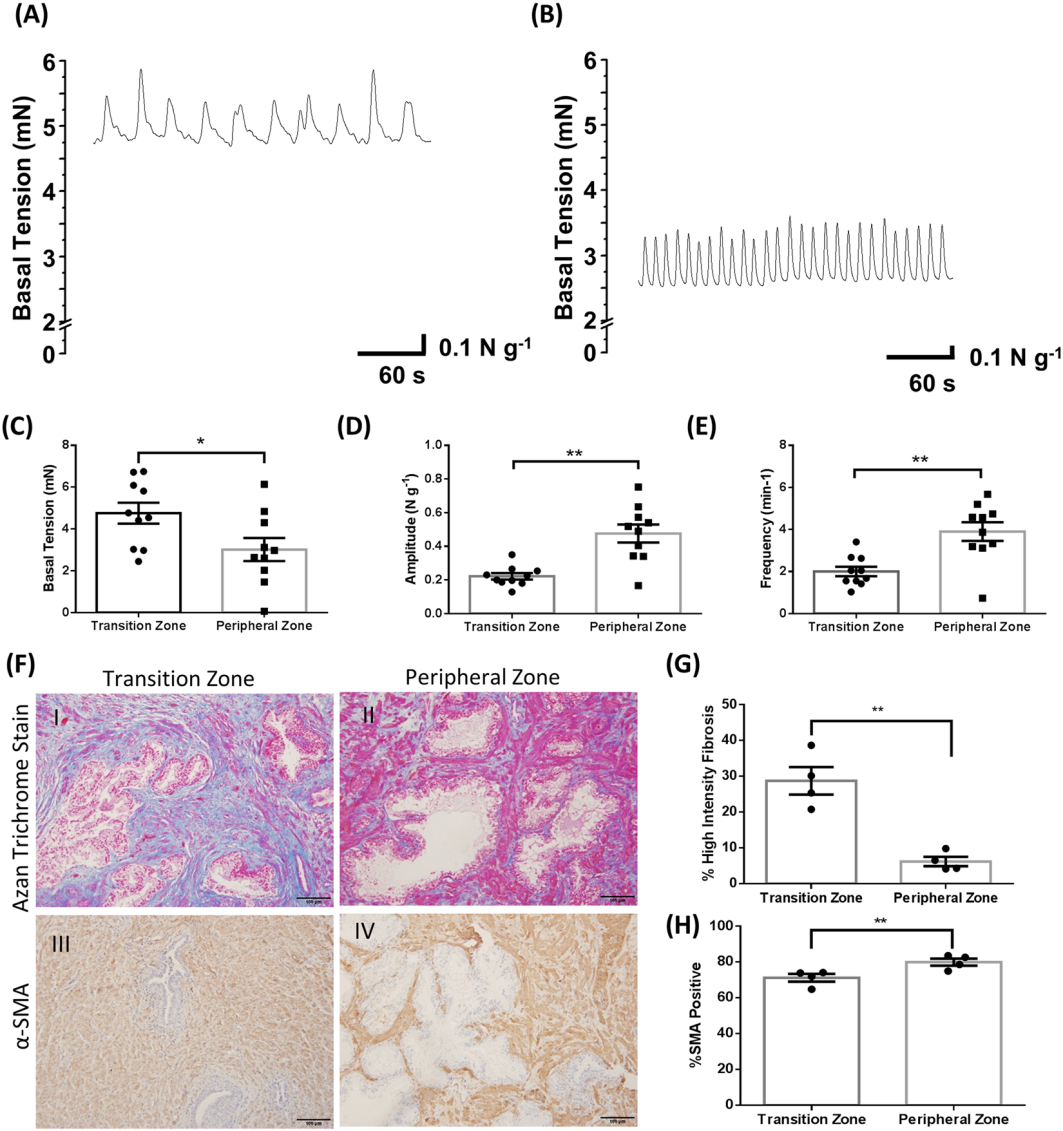
Myogenic contractility is significantly different in Transition *versus* Peripheral zones of the human prostate. Representative traces of spontaneous activity in the (**A**) Transition Zone and (**B**) Peripheral Zone, and the average (**C**) resting basal tension, (**D**) amplitude, and (**E**) frequency of spontaneous contractions in the transition zone in comparison to the peripheral zone of matched patients (Student’s paired t-test, n = 10, * indicates *p* < 0.05, ** indicates *p* < 0.01). (**F**) representative images of I) & II) Azan Trichrome Staining and III) & IV) α-Smooth Muscle Actin (α-SMA) from the I) & III) Peripheral and II) & IV) Transition zone of a same matched patient. (**G**) Percent of positive stromal pixels corresponding to strong collagen staining in sections stained with Azan Trichrome (Student’s paired t-test. N = 4, **indicates *p* < 0.01) (**H**) Percentage of positive staining for α-SMA to the total stromal area (Student’s paired t-test. N = 4, **indicates *p* < 0.01).

Tension recordings were used to assess myogenic contractility in patient TZ and PZ tissue specimens (Fig. 1A and B, representative traces from TZ and PZ tissues from the same individual patient). TZ specimens demonstrated a higher basal tension (Fig. 1C) compared to PZ tissues, while the amplitude (Fig. 1D) and frequency of contractions (Fig. 1E) were significantly greater in PZ specimens. The difference in contractility between the zonal regions was associated with differences in tissue composition that is evident in the pathology of matched TZ and PZ specimens from the same patients. Azan trichrome staining showed zonal differences in the distribution of glands and smooth muscle cells within the stroma (Fig. 1F), and increased fibrosis in TZ tissue (Fig. 1G). Sections stained for α-SMA (Fig. 1F), revealed that PZ tissue possesses a significantly greater number of α-SMA positive cells than patient-matched TZ specimens (Fig. 1H). Application of blockers of neural transmitters, Tetrodotoxin (TTX; 1 µM, n = 5) or Atropine (1 µM, n = 6), did not have any effect on myogenic contractile parameters (Supplementary Figure 1).

### Myogenic tone is significantly greater in the transition zone of men with clinically diagnosed BPH

**Figure 2:**
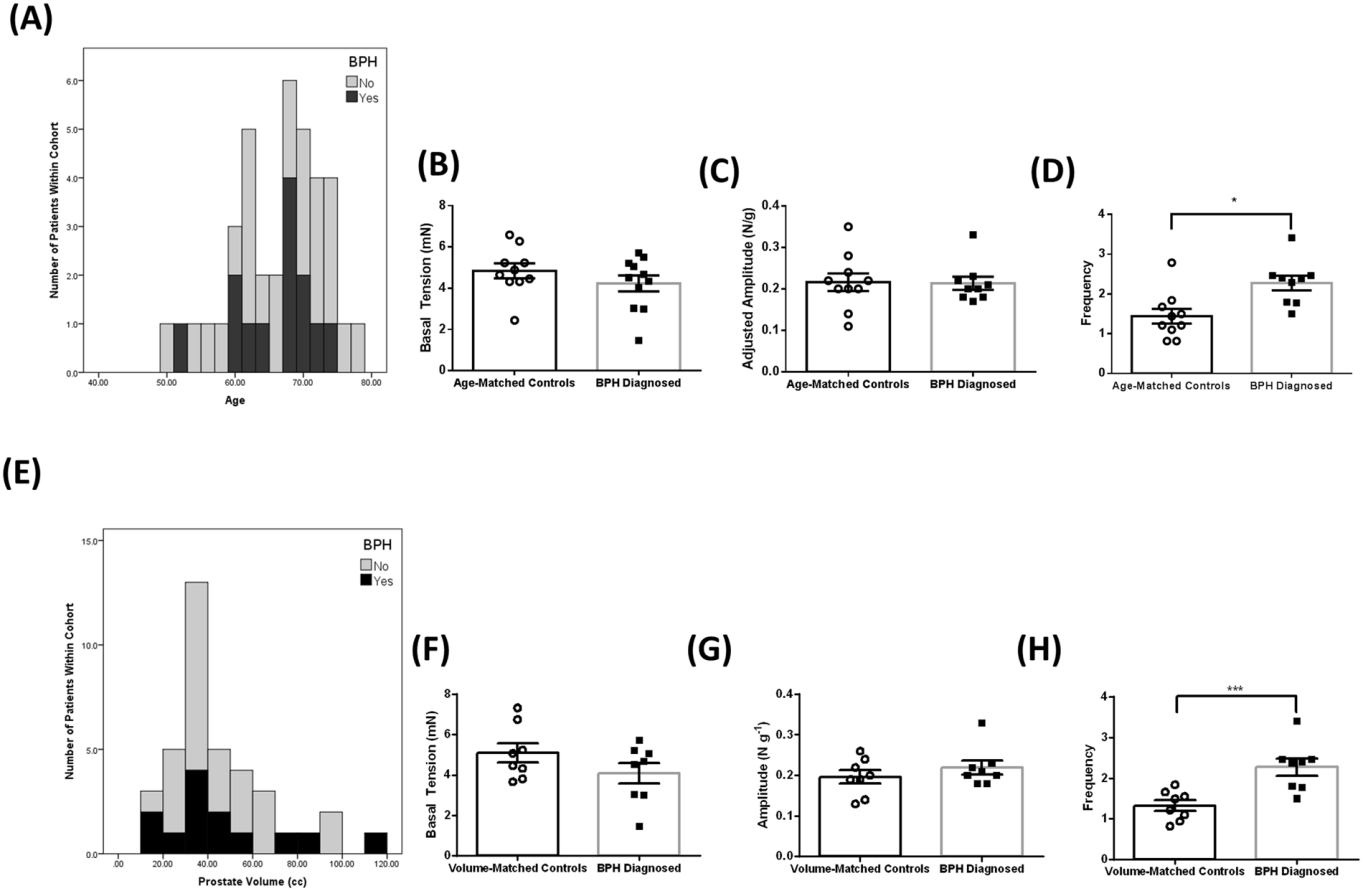
The frequency of myogenic contractions is significantly higher in Transition Zone specimens from men with BPH compared to age and volume matched controls. Frequency distribution histograms of BPH diagnosed men within the patient cohort by (**A**) Age and (**B**) Prostate Volume. The average (**C**,**F**) basal tension (mN), (**D**,**G**) amplitude and (**D**,**H**) frequency of spontaneous contractions within the TZ of men with clinically diagnosed BPH compared to age (**B**,**C**,**D**) and prostate volume (**F**,**G**,**H**) matched controls (Student’s unpaired t-test, n = 9, * indicates p < 0.05, *** indicates p < 0.001).

We established a clinically diverse cohort of tissues from 38 men (details in Table 1). Within this cohort, there was a subset of 13 patients who had clinical BPH associated with LUTS (Fig. 2A and E) that was retrospectively identified from clinical records. This subgroup was used to test if myogenic tone is altered in TZ tissue from men with BPH. We compared tension recordings of myogenic contractions in tissue from the men with clinical BPH to age-matched (similar age ± 2 years, not receiving pharmacotherapy at the time of prostatectomy, prostate volume ≤40cc; Fig. 2A–D), or to prostate volume-matched controls (similar prostate volume ± 5cc, not receiving pharmacotherapy at the time of prostatectomy; Fig. 2E–H). Four (4) men from the BPH cohort were excluded from analysis based on a Grubb’s test, and previous treatment with pharmacotherapies, resulting in the BPH cohort consisting of n = 9. The frequency of myogenic contractions was significantly (*p* < 0.05) greater in TZ specimens collected from men with clinically diagnosed BPH when compared to both age (Fig. 2D) and prostate volume-matched (Fig. 2H) controls. The frequency of contractions in BPH diagnosed patients was 157.63% and 171.97% greater than age and prostate volume-matched controls, respectively. Basal tension and amplitude of contractions was not significantly different in men with BPH when compared to age (Fig. 2B and C) or volume (Fig. 2F and G) matched controls. Overall these results show the frequency of myogenic contractions is greater in men with BPH.

**Table 1:** Characteristics of the BPH Patient Cohort.

Demographic	Number (% of cohort)	Mean (±SD)	Range
Total Patients	38 (100%)		
Clinical BPH Diagnosis	13 (34.2)		
LUTS reported	16 (42.1)		
LUTS and BPH	8 (21.1)		
Age		65.5 (6.6)	50–77
Prostate Volume (cc)		44.7 (22.0)	12–144
Gleason Score		7 (N/A)	6–9

### Tamsulosin attenuates myogenic contractility in the human prostate with responsiveness correlating to older patients and larger prostate volumes

**Figure 3:**
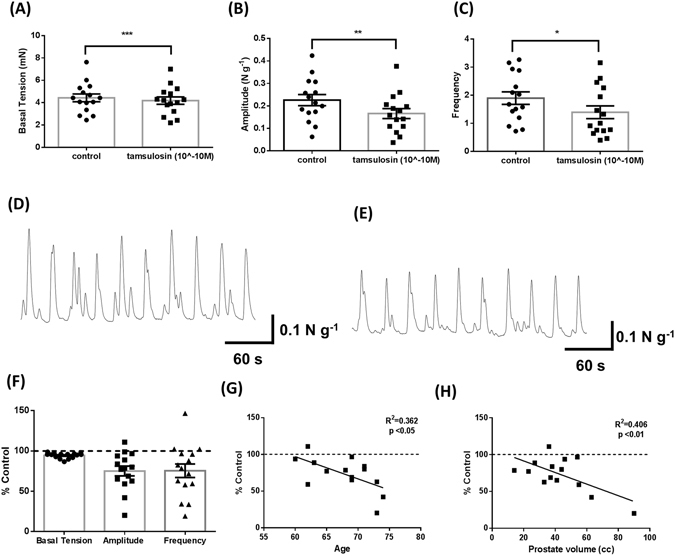
Tamsulosin (10^−10^M) decreases myogenic contractility in Transition Zone specimens, with larger response correlating to increased age and prostate volume. (**A**) Basal tension, (**B**) amplitude, and (**C**) frequency of contractions pre and post tamsulosin (0.1 nM) (Student’s paired t-test, n = 15, * indicates p < 0.05, ** indicates p < 0.01, *** indicates p < 0.001), (**D**) representative trace showing pre-treatment control activity and (**E**) representative trace showing treatment response. (**F**) Contractile Parameters following administration of Tamsulosin were converted to a percentage of the original (untreated) contractile parameters. Correlation between (**G**) age and (**H**) prostate volume and percentage decrease in amplitude of spontaneous contractions (Linear Regression Analysis, p < 0.05 considered significant).

To determine if current therapies for BPH modulate myogenic tone we tested the response to α1_A_-adrenoreceptor antagonist Tamsulosin *in vitro* in an organ bath model system. Tamsulosin (0.1 nM) significantly reduced the basal tension, amplitude and frequency of myogenic contractions in TZ specimens following incubation for 30 minutes (Fig. 3A,B and C, respectively; representative trace Fig. 3D and E). Regression analysis was performed using clinical parameters obtained from retrospective analysis of patient details against the percentage of the control activity (% control), with 0% indicating complete abolishment of contractile activity, and 100% indicating contractility remained unchanged following treatment (Fig. 3F). Age and prostate volume both significantly (P < 0.05) negatively correlated with the % control change in the amplitude of contractions (Fig. 3G and H, respectively). Overall, demonstrating a greater efficacy of Tamsulosin at reducing myogenic activity in older men and/or those with larger prostate volumes.

### Sildenafil decreases myogenic contractility with responsiveness significantly correlated to younger patients

**Figure 4:**
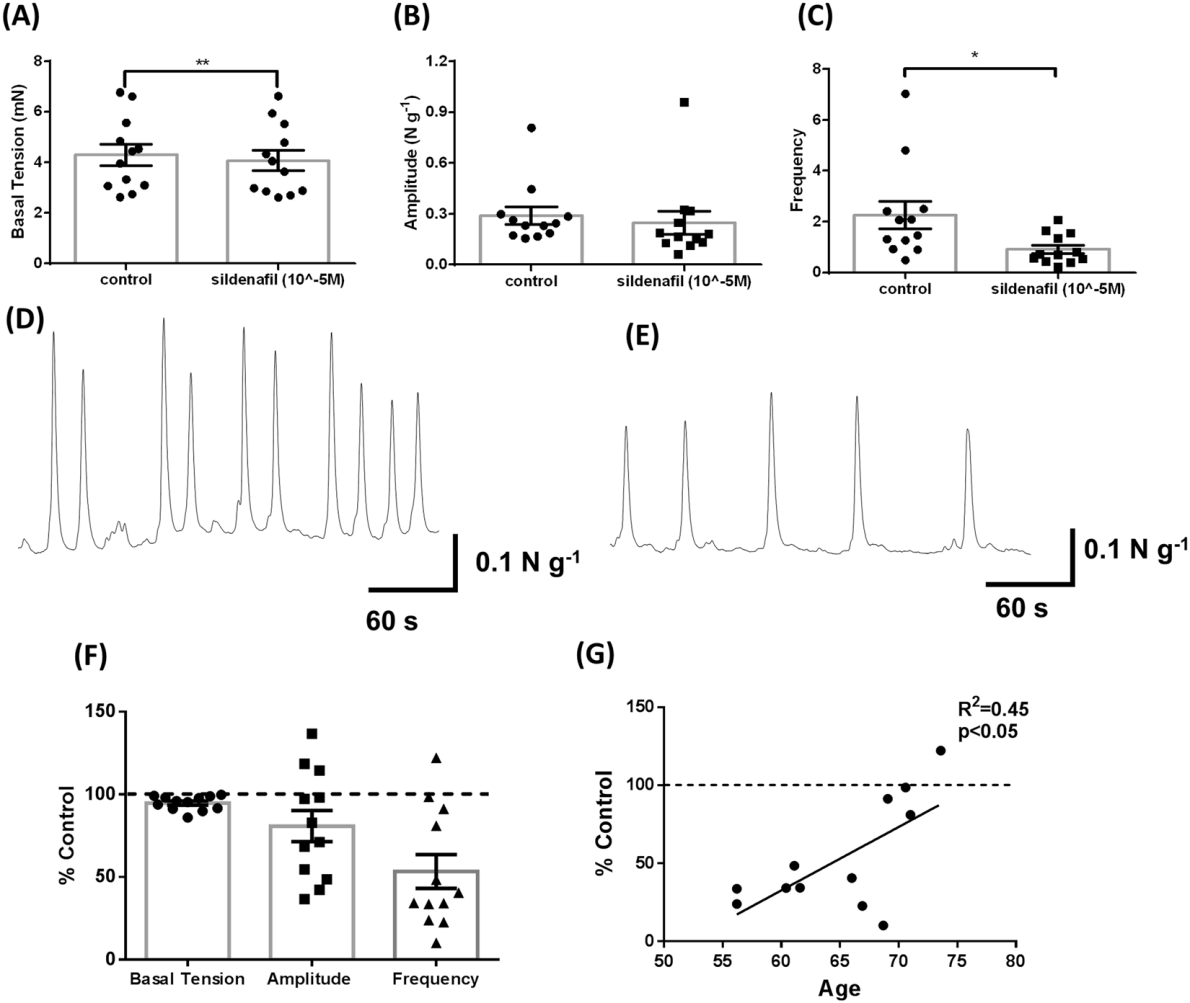
Sildenafil (10^−5^ M) significantly decreases basal tension and frequency of myogenic contractility within the human prostate Transition Zone. (**A**) Basal tension, (**B**) amplitude, and (**C**) frequency of contractions pre and post sildenafil (10 µM) (Student’s paired t-test, n = 12, * indicates p < 0.05, ** indicates p < 0.01), (**D**) representative trace showing pre-treatment control activity and (**E**) representative trace showing treatment response. (**F**) Contractile Parameters following administration of sildenafil were converted to a percentage of the original (untreated) contractile parameters. Correlation between (**G**) age and percentage decrease of the frequency of spontaneous contractions (Linear Regression Analysis, p < 0.05 considered significant).

PDE5-Is appear to reduce LUTS secondary to BPH, although the mechanism of action in the prostate is unclear. To test if the PDE5-I Sildenafil altered myogenic contractility we conducted similar *in vitro* organ bath studies. Sildenafil (10 µM) significantly decreased the basal tension and frequency of myogenic contractions in TZ specimens following incubation for 30 minutes, while amplitude was unaffected (Fig. 4A–C; representative traces Fig. 4D and E). There was a large interpatient variability associated with the decrease in frequency in response to Sildenafil, with the % control ranging from 10.2–122.2% (Fig. 4F). Regression analysis was used to compare % control of the frequency of contractions with age and demonstrated overall a significant positive correlation between % control and age (Fig. 4G).

Immunohistochemistry was conducted to assess the presence and distribution of PDE5. PDE5 expression was observed within the stroma, but not the epithelium (Supplementary Figure 1). In 80% (4/5) patients, there was also higher expression of PDE5 in the TZ compared to patient matched PZ specimens when semi-quantitatively assessed (Supplementary Figure 1). PKG-1, a downstream protein of the cGMP signalling pathway and the most important protein involved in relaxation, was also assessed as a positive internal control for cGMP pathway expression. PKG-1 was strongly expressed in the stroma, with no epithelial staining. There was also extremely high expression of PKG-1 in the vasculature of the prostate, whereas PDE5 expression in vessels was lower (Supplementary Figure 2). Importantly, this vascular localization of PDE5/PKG1 is well known for other tissues and peripheral blood vessels do not contract spontaneously, thus the PDE5 expression in prostatic vessels cannot influence the contractility observed in our organ bath studies. Collectively, these data highlight the potential of PDE5-Is to alleviate LUTS, particularly in younger patients.

## Discussion

Our study is the first to describe and characterize spontaneous myogenic contractility in the human prostate gland. Using a cohort of 38 men we show that the frequency of myogenic contractions is increased in men with clinical BPH. Additionally, we also show that the features of myogenic tone can be selectively attenuated by two current BPH therapeutics, Tamsulosin and Sildenafil. Although there is substantial interpatient variability, the response to Tamsulosin and Sildenafil is correlated with specific patient parameters (age, prostate volume).

These novel findings have significant implications for improving patient outcomes for BPH similar to what has occurred in the bladder. Since the initial observation by Brading and colleagues that there is a myogenic basis for the overactive bladder^13^ there has been a significant shift in the field of bladder research into understanding the mechanisms underlying myogenic tone in bladder contractility, and developing therapeutics targeting this component. The myogenic hypothesis is a current rationale for the pathophysiology of overactive bladder disease^16^. Recently, Botulinum toxin A (BoNT-A) was described as attenuating myogenic contractions of the rat bladder^17^, and is currently used as a treatment for overactive bladder and detrusor overactivity^18^. We now demonstrate that human prostate contractility also possesses a myogenic component. The myogenic origin of the contractions is evident as application of TTX (which prevent the release of neurotransmitters from the synaptic cleft on the neuronal axon terminal^19^) or Atropine (a Muscarinic Receptor antagonist), did not attenuate any of the myogenic contractile parameters. Therefore, these contractions are of a myogenic, not neurogenic, origin.

BPH arises in the TZ of the human prostate, and we found that the frequency of myogenic contractions in the TZ was significantly higher in men with clinically diagnosed BPH when compared to both age and prostate-volume matched controls. Prostatic smooth muscle vigorously contracts during ejaculation in order to expel prostatic fluids from the glands of the prostate into the ejaculate^12^. This is regulated by the Sympathetic Nervous System (SNS), which releases noradrenaline acting on α1-adrenoreceptors to generate the neurogenic contraction. α1-adrenoreceptors are the predominant SNS receptor within the human prostate^20^, and have elevated expression in BPH diagnosed men^21, 22^. Consequently, alpha blockers (e.g Tamsulosin) have been one of the mainstay therapies for BPH for decades, although they have unpredictable efficacy, and are associated with undesirable side effects^4^. Myogenic tone is fundamentally different in that contractions are generated independently of stimulus from the SNS, occur consistently, and are of a smaller magnitude. As the frequency of these contractions is upregulated in men with clinically diagnosed BPH, we hypothesize that an ideal pharmacotherapy will target both neurogenic and myogenic tone. Significantly, we found that Tamsulosin diminished myogenic activity in TZ, with a reduction in all parameters (basal tension, amplitude and frequency). Further, subsequent regression analysis revealed an association between specific clinical parameters and the response to Tamsulosin in terms of amplitude and frequency. Specifically, older patients, or patients with larger prostate volumes, demonstrated a greater reduction in the amplitude of myogenic contractions in response to Tamsulosin. Along with the increased density of α1-adrenoreceptors in the human prostate with age^23^, our data provide further rationale for the use of α1-adrenoreceptor antagonists specifically in older men (>70 years), or those with larger prostates (>40cc).

PDE5 inhibitors were recently approved for the treatment of BPH, and are included in the guidelines from both the AUA and EAU regarding therapeutics for the treatment of this disease^4, 24^. Initially used to treat erectile dysfunction, there is little evidence of the mechanism of action on the human prostate gland^24^ although patients reported significant reductions in IPSS score^25^. Our *in vitro* studies show a direct effect of Sildenafil, significantly reducing basal tension and frequency of myogenic contractions. The decrease in frequency was variable between patients but was inversely correlated with patient age and older patients (>70) demonstrated little to no response to Sildenafil. This observation is supported by a recent meta-analysis that found older patients experienced limited improvement in IPSS scores with PDE5-Is compared to younger men^25^, as well as the observed PDE5 expression levels in prostate tissue, which was higher in younger patients (Supplementary Figure 1). Collectively these data suggest that the use of PDE5 inhibitors for BPH is more suited to younger men.

One of the major challenges with a study of this type is obtaining viable primary human prostate tissue for tension recordings. Transurethral Resection of the Prostate (TURP) is the most common surgical procedure for men with BPH. However the tissue is unsuitable for investigation for two reasons. Firstly, TURP is commonly performed after treatment failure and cannot provide treatment naïve specimens. Secondly, the tissue is frequently physically damaged in the process, rendering it unable to be used for subsequent studies. We overcame these issues by obtaining tissue from men undergoing radical prostatectomy for localised prostate cancer and from their clinical records we identified men with LUTS and clinical BPH. We used tissue from patients with low volume tumours in PZ from whom we could obtain TZ tissues for investigation. This approach, however, limits clinical details regarding LUTS and BPH symptomology, with only two patients in the cohort completing the IPSS questionnaire prior to surgery.

Overall, BPH is an extremely prevalent disease with a high economic and social impact. Current therapeutics lack overall and consistent efficacy, while predicting patient response is currently impossible. Our study provides evidence that myogenic (spontaneous) activity is associated with BPH within the human prostate. Frequency of myogenic contractions is elevated in men with BPH, and is diminished using current therapeutics (Tamsulosin and Sildenafil). Importantly, this response significantly correlated with patient age and prostate volume, with Tamsulosin demonstrating greater efficacy in tissues from older men and those with larger prostate volumes, while Sildenafil was most effective in tissues from younger men. Collectively, these data highlight that therapeutics that modulate myogenic tone, when applied based on patient characteristics such as age, may yield improved and predictable clinical outcomes for patients with BPH.

## Materials and Methods

### Patient Cohort

Human prostatic tissue was collected with informed written consent from patients and approval from the Cabrini Human Research Ethics Committee (13-14-04-08), Epworth HealthCare Human Ethics Committee (53611) and Monash University Human Research Ethics Committee (2004/145). All experiments were performed in accordance with relevant guidelines and regulations. Tissue was collected from 38 men undergoing radical prostatectomy for the treatment of low to medium grade prostatic cancer (Gleason Score ≤ 7). A rigorous tissue selection procedure was employed to minimize any possible influence of the tumour on the non-malignant tissue. Only patients with a single tumour foci located in the PZ were selected, and, secondly, only those with a very low tumour volume as a percentage of the prostate weight (under 10%) were utilized. Patient clinical details are shown in Table 1.

Following prostatectomy, pieces of non-malignant prostatic tissue were cut from the transition (TZ) and peripheral (PZ) zone of the prostate by a certified pathologist, and placed in chilled Physiological Saline Solution (PSS) for transportation to the research facilities. Tissue was prepared by micro-dissection into preparations (2mm × 5mm) for organ bath studies, or fixed in formalin (10%) for histological analysis. Patient clinical records were retrospectively accessed through physicians, hospitals, and diagnostic laboratories. Clinical BPH was diagnosed by the surgeons prior to radical prostatectomy, and was defined based on the grade of Intravesical Prostatic Protrusion (IPP), and the presence of LUTS. Patients with prior pharmacological treatment for BPH were excluded from the cohort.

### Organ Bath Studies

Tension recordings were obtained from TZ and PZ specimens as previously described^26–28^. An initial tension of 25mN was applied to the tissue, and left to equilibrate for 60 minutes. The ‘basal tension’ describes the tension of the tissue following this equilibration period. This tension was not re-adjusted to a predefined baseline to allow spontaneous contractions to emerge. Following the equilibration period, the preparation was incubated with either Tamsulosin (0.1 nM), Sildenafil (10 μM), Atropine (1 µM) or tetrodotoxin (TTX:1 µM), prepared in fresh Physiological Saline Solution (PSS), for a period of 30 minutes (see supplementary methods for details of all chemicals and reagents). Following a washout period, the tissue was challenged with a potassium chloride solution (20 mM) to assess tissue viability and reliably induces a robust contraction in all viable preparations. Preparations were subsequently weighed following the completion of experiments.

Analysis of tension recordings focused on three parameters: basal tension (the inherent tension within the tissue; mN), as well as amplitude and frequency (strength and speed of contractions; N/g and min^−1^, respectively) as previously reported^26, 29^. These parameters are as used in previous studies to define myogenic tone^26–28^. Chart Pro ® v 5.5.6 was used to analyse the tension recordings, with indicated parameters measured for 5 consecutive responses and averaged before and during incubation with Tamsulosin (0.1 nM) or Sildenafil (10 μM).

### Immunohistochemistry and tissue stains

Sections were de-paraffinised and rehydrated through a series of xylene and graded ethanol solutions. Sections were placed in 1.2% H_2_O_2_ for 30 minutes to block endogenous peroxidase activity, and were incubated with the primary antibodies; PKG-1 (1:1000, ADI-KAP-PK005-F, Enzo Life Sciences), PDE5A (1:500, FabGennix) and α-Smooth Muscle Actin (α-SMA) (1:10,000, Sigma), diluted in PBS with 0.2% BSA and 0.1% sodium azide for 24 hours at 4 °C. Immunoreactivity was visualized using the DAKO EnVision kit (DAKO, Hamburg, Germany) followed by the nickel-glucose oxidase approach^30^. Antigen retrieval was performed prior to incubation with the primary antibody for sections incubated with the α-SMA and PDE5A antibody by boiling citrate-buffer (pH 6.0) in a microwave (9 minutes at 700 W followed by 15 minutes at 450 W). Negative controls were performed by omitting the primary antibody.

Slides were scanned using an Aperio ScanScope scanner and Aperio ImageScope software was used to visualize and define stromal regions of the tissue, excluding glandular regions. The Aperio Color Deconvolution v9 algorithm was used to quantify the percent of positive pixels corresponding to strong collagen staining, while the Positive Pixel Count v9 algorithm with default parameters was used to determine α-SMA cell counts. Blinded studies were conducted using three (3) naïve viewers, who were instructed to score the intensity of staining using the following criteria; (−) no staining, (+) low staining, and (++) high staining.

### Chemicals and Reagents

Physiological saline solution (PSS) was composed of 2.5 mM CaCl_2_, 11 mM D-Glucose, 5 mM KCl, 120 mM NaCl, 25 mM NaHCO_3_, 1.2 mM KH_2_PO_4_, 1.2 mM MgSO_4_, bubbled with a 95% O_2_: 5% CO_2_ gas mixture to maintain a pH of 7.3-7.4. Sildenafil (Sigma, St Louis, MO, USA) was dissolved in dimethyl sulfoxide (DMSO, Sigma, St Louis, MO, USA) to create a stock solution (10 mM). Tamsulosin (Yamanouchi Pharmaceutical Co. Ltd, Tokyo, Japan; gift from Assoc. Prof. J. N. Pennefather) was dissolved in distilled water to create a stock solution (0.01 mM). Stock solution was diluted in PSS to the required concentrations and bubbled with a 95% O_2_: 5% CO_2_ gas mixture prior to use.

### Statistical Analysis

Patient-matched TZ and PZ specimens were compared using a Student’s paired t-test. Student’s unpaired t-tests were used to compare contractile parameters between groups of men within the cohort. Treatment with a single pharmacotherapy was compared using a Student’s paired t-test with GraphPad Prism version 6.04 for Windows (GraphPad Software, La Jolla California USA). An Extreme Studentized Deviate (ESD) test with an Alpha level of 0.05 was used to exclude outliers. Multi-linear regression analysis was used to correlate clinical records with the percentage response to pharmacotherapy treatment using IBM SPSS Statistics for Windows, Version 22.0. All error bars on all graphs represent the Standard Error of the Mean (SEM).

## Electronic supplementary material

Supplementary figures
