# Supplementary material for: Age Related Differences in Responsiveness to Sildenafil and Tamsulosin are due to Myogenic Smooth Muscle Tone in the Human Prostate
Source: Sci Rep. 2017 Aug 31;7:10150. doi: 10.1038/s41598-017-07861-x (PMC5578961; doi:10.1038/s41598-017-07861-x)
Supplement: Supplementary file 1 — Supplementary figures [file 41598_2017_7861_MOESM1_ESM.doc]

**Age Related Differences in Responsiveness to Sildenafil and Tamsulosin are due to Myogenic Smooth Muscle Tone in the Human Prostate**

**Authors:** Sophie N. Lee^2^, Basu Chakrabarty^1^, Brad Wittmer^1,2^, Melissa Papargiris^2^, Andrew Ryan^3^, Mark Frydenberg^4,5^, Nathan Lawrentschuk^6^, Ralf Middendorff^7^, Gail P. Risbridger^2^, Stuart J. Ellem^2†^ & Betty Exintaris^1†^*^*^*

**Affiliations:**

^1^ *Drug Discovery Biology, Monash Institute of Pharmaceutical Sciences, Melbourne, Victoria, Australia*

^2^ *Department of Anatomy and Developmental Biology, Biomedicine Discovery Institute, Monash University, Clayton, Victoria, Australia*

^3^ *TissuePath, Melbourne, Victoria, Australia*

^4^ *Department of Surgery, Monash University, Melbourne, Victoria, Australia*

^5^ *Australian Urology Associates, Melbourne, Victoria, Australia*

^6^ *Department of Surgery, Austin Health, University of Melbourne, Melbourne, Victoria, Australia*

^7^ *Justus-Liebig-University Giessen, Institute of Anatomy and Cell Biology, Giessen, Germany*

† = These authors contributed equally to this work


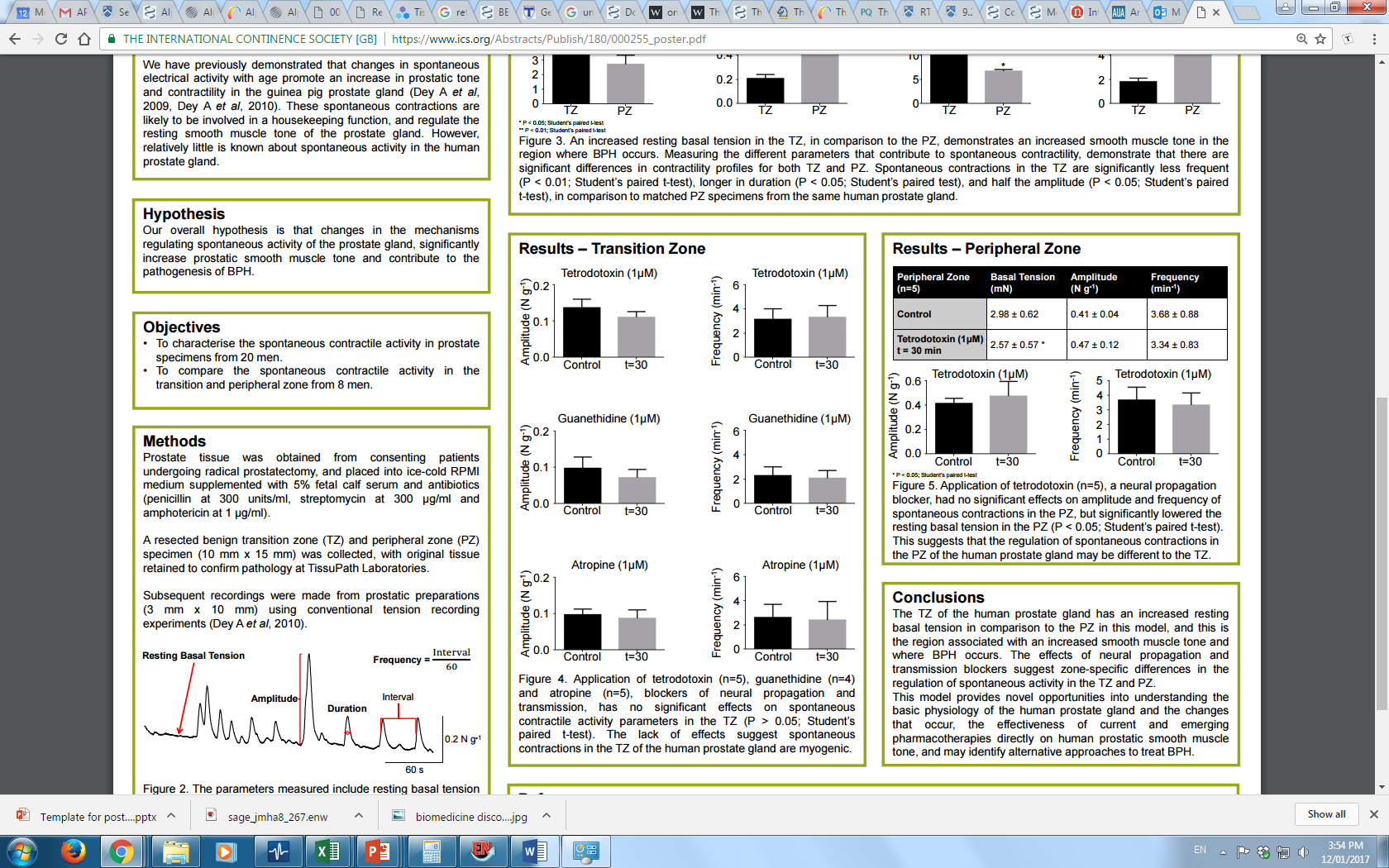

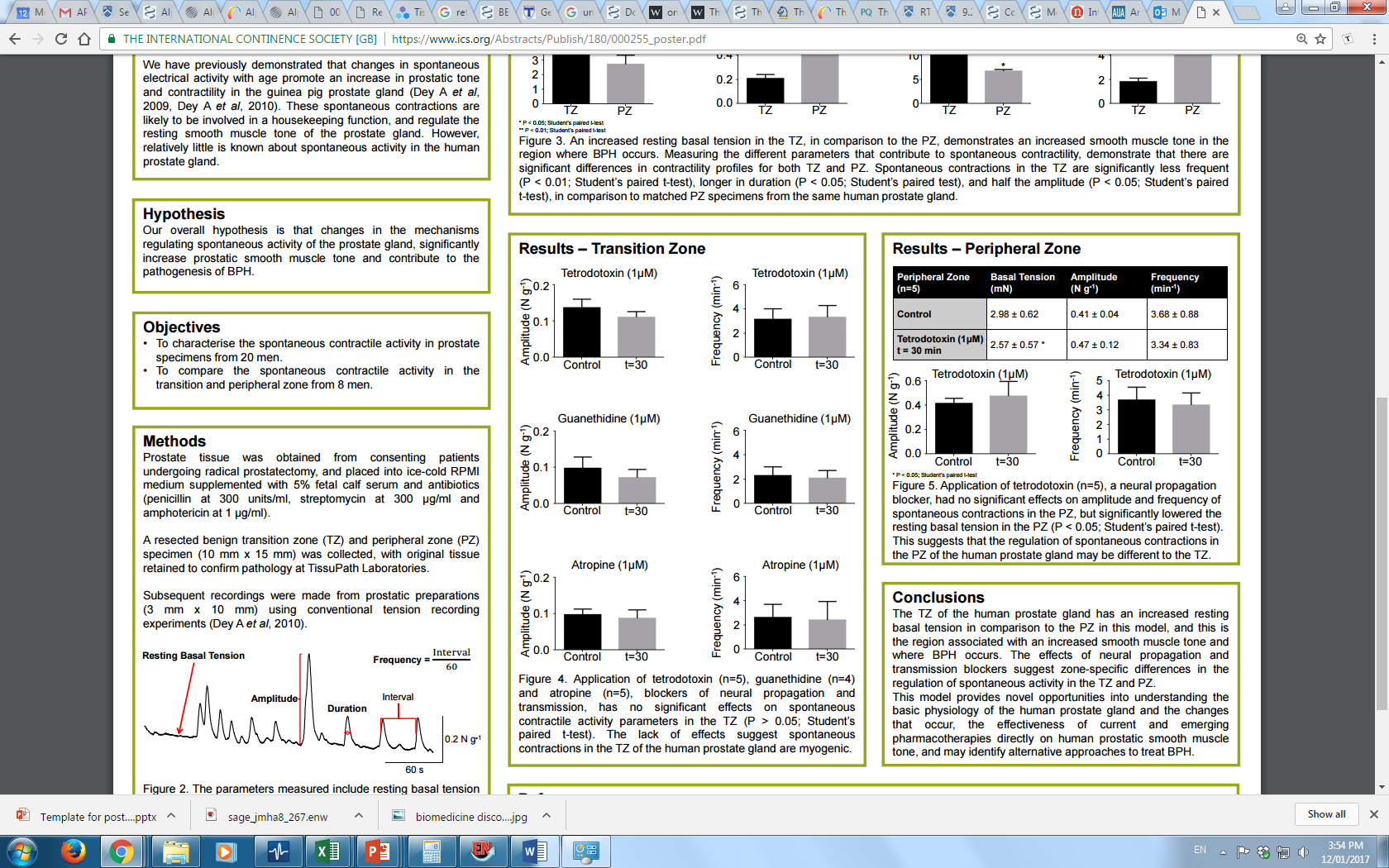


**Supplementary Figure 1: Application of blockers of neuronal propagation did not attenuate myogenic contractility parameters** (A) Effect of Tetrodotoxin (1μM) on the (I) amplitude and (II) frequency of myogenic contractions in TZ specimens (n= 5) (B) Effect of Atropine (1μM) on the (I) amplitude and (II) frequency of myogenic contractions in TZ specimens (n= 5)

**(A)**

**(I)**

**(II)**

**(B)**

**(I)**

**(II)**


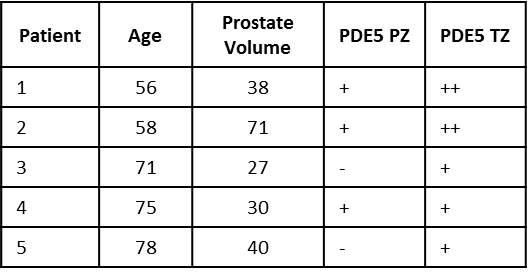

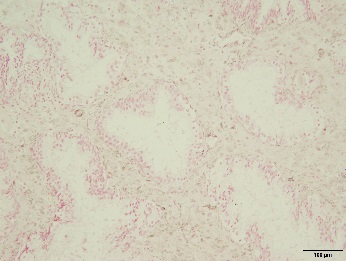

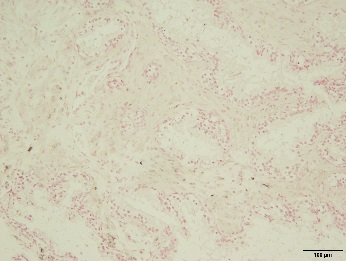

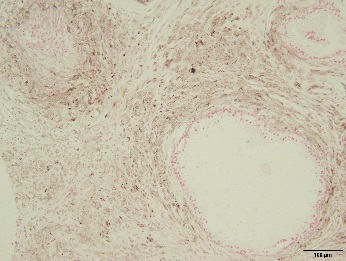

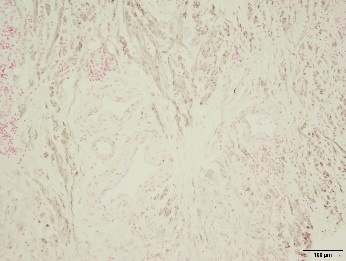


**PKG-1**

**PDE5A**

**Peripheral Zone**

**Supplementary Figure 2: Inter- and intra- patient variability in the distribution of PDE5 and PKG-1 as assessed by immunohistochemistry** (A) Expression of PKG-1 (I & II) and PDE5 (III & IV) in the Transition Zone (I & III) or Peripheral Zone (II & IV) of a matched patient. (B) Blinded assessment of immunohistochemical PDE5 staining intensity in a subset of five (5) patients (- no expression, + low expression, ++ high expression)

**(A)**

**(B)**

**I**

**II**

**Transition Zone**

**III**

**IV**
